# Development of a Smart Ball to Evaluate Locomotor Performance: Application in Adolescents with Intellectual Disabilities

**DOI:** 10.3390/s20185444

**Published:** 2020-09-22

**Authors:** Wann-Yun Shieh, Yan-Ying Ju, Yu-Chun Yu, Steven Pandelaki, Hsin-Yi Kathy Cheng

**Affiliations:** 1 Department of Computer Science and Information Engineering, College of Engineering, Chang Gung University, No. 259, Wen-Hwa 1st Road, Kwei-Shan, Tao-Yuan 333, Taiwan; wyshieh@mail.cgu.edu.tw; 2 Department of Physical Medicine and Rehabilitation, Chang Gung Memorial Hospital, 5 Fu-Hsing Street, Kwei-Shan, Tao-Yuan 333, Taiwan; 3 Department of Adapted Physical Education, National Taiwan Sport University, No. 250, Wen-Hwa 1st Road, Kwei-Shan, Tao-Yuan 333, Taiwan; yanju@ntsu.edu.tw; 4 Taoyuan Municipal Taoyuan Special School, No. 10, Deshou St., Taoyuan Dist., Tao-Yuan 330, Taiwan; yuechung@ms.tsad.tyc.edu.tw; 5 Department of Informatics Engineering, De La Salle Catholic University, Manado 95001, Indonesia; spandelaki@unikadelasalle.ac.id; 6 Graduate Institute of Early Intervention, College of Medicine, Chang Gung University, No. 259, Wen-Hwa 1st Road, Kwei-Shan, Tao-Yuan 333, Taiwan

**Keywords:** intellectual disabilities, locomotor performance, coordination, smart ball

## Abstract

Adolescents with intellectual disabilities display maladaptive behaviors in activities of daily living because of physical abnormalities or neurological disorders. These adolescents typically exhibit poor locomotor performance and low cognitive abilities in moving the body to perform tasks (e.g., throwing an object or catching an object) smoothly, quickly, and gracefully when compared with typically developing adolescents. Measuring movement time and distance alone does not provide a complete picture of the atypical performance. In this study, a smart ball with an inertial sensor embedded inside was proposed to measure the locomotor performance of adolescents with intellectual disabilities. Four ball games were designed for use with this smart ball: two lower limb games (dribbling along a straight line and a zigzag line) and two upper limb games (picking up a ball and throwing-and-catching). The results of 25 adolescents with intellectual disabilities (aged 18.36 ± 2.46 years) were compared with the results of 25 typically developing adolescents (aged 18.36 ± 0.49 years) in the four tests. Adolescents with intellectual disabilities exhibited considerable motor-performance differences from typically developing adolescents in terms of moving speed, hand–eye coordination, and object control in all tests.

## 1. Introduction

The American Association of Intellectual and Developmental Disabilities (AAIDD) reported that approximately 1–3% of the global population has an intellectual disability [1]. Intellectual Disability (ID), also known as mental retardation, is characterized by significant limitations in cognitive functioning and skills during the developmental period (before the age of 18) [2,3]. These limitations cause poor visual and motor coordination, limited precision of movements, and difficulties in learning new forms of activities, when compared with typically developing (TD) adolescents [4,5,6].

Among cognition and skill impairments, locomotor performance is a valuable indicator to distinguish motor dysfunction in adolescents with ID and TD adolescents. Good locomotor performance, such as moving the body or changing direction and position quickly to finish a task while maintaining balance, requires numerous locomotor skills, such as balance, coordination, speed, reflex, strength, endurance, and stamina. Disorders of these skills can affect gait, movement stability, object control, and numerous other aspects [7,8]. Delayed development of locomotor performance has been demonstrated to be associated with poor cognitive function in daily life [6].

Locomotor performance can be evaluated using regular gross motor tests that evaluate walking, running, or other specific motions [7,9,10,11]. However, some of these tests do not display significant differences between TD and adolescents with ID. Iosa et al. [7] illustrated that, unless the adolescents have multiple disabilities, no significant difference is observed between these two groups in a straight-line walking test. Shieh et al. [10] reported that adolescents with ID only present a significant difference in locomotor performance when compared with adolescents with TD when the test is more challenging and requires more upper or lower extremity muscle control and stability. Furthermore, most participants with ID fail to follow the instructions if the test does not involve a familiar task or cannot retain attention. Consequently, tracking the locomotor performance of people with ID is challenging.

Ball-related activities are appealing and familiar tasks for individuals with ID. The skills involved in ball activities, such as throwing, catching, passing, and dribbling, require hand–eye coordination, foot–eye coordination, and body balance. Furthermore, these activities are entertaining, easy to learn, and promote interaction and physical activities. Therefore, they are often employed by schools and organizations in group activities and physical exercises. Moreover, ball activities are useful in locomotor performance evaluation. Most performance evaluations of motor activities require participants’ full attention. Since adolescents with ID usually have deficits in concentration, more time is needed to complete one-on-one evaluations. An efficient yet objective evaluation tool is needed in numerous areas, such as special education schools where numerous adolescents with ID must be evaluated simultaneously at the start of each semester. Evaluating demanding actions in a ball-related test (e.g., dribbling the ball while running) can discriminate the physical performance levels between adolescents with ID and TD adolescents more effectively than simple tests of walking or running [12,13].

Studies evaluating ball activities in adolescents with ID are limited and most commonly known approaches rely on expert scoring, such as the Peabody Developmental Motor Scales, Second Edition (PDMS-2) [14]. The testing items in PDMS-2 include object manipulation skills (e.g., catching, throwing, and kicking a ball). The Preschooler Gross Motor Quality Scale (PGMQS) also includes ball-related evaluations [15], but it was designed for preschool children. The scores of these tests are determined by medical professionals with scores ranging from 0–5 points per item. However, the scoring results only represent an overall description of ball skill performance. Detailed performance parameters or locomotor patterns cannot be objectively measured using manual scoring and the reports are not objective and automatic. Therefore, wearable devices have been adopted [9,16,17,18,19].

With the rapid development of inertial sensor technology, wearable devices have become convenient. Portable tools can be applied in daily life and unrestricted-distance measurements to raise awareness of locomotor abnormalities throughout childhood and adulthood [16]. Most wearable devices contain a triaxial accelerometer and a gyroscope. The accelerometer measures the acceleration of an object along its movement trajectory, and the gyroscope measures an object’s rotational angular velocity around the roll, pitch, and yaw axes [18]. These sensors can be mounted on different parts of the body to measure the movement of the limbs or the trunk without excessive restrictions. However, wearable devices do not satisfy all measurement requirements. For instance, a wearable sensor cannot measure the real outcome of ball-related skill tests, such as the height of ball throwing, the catching of the ball after throwing, or dribbling the ball along an indicated path.

A smart ball with embedded inertial sensors is proposed in the present study to compensate for the limitations of wearable devices. The embedded sensors included an accelerometer and a gyroscope, which can sense the force of gravity on the ball and the rotation momentum of the ball around three axes. The sensor signals were transmitted from the ball to a mobile device through Bluetooth transmission. Therefore, any tests with this ball can be completed wirelessly. The signals from the ball were highly informative because the ball-related skill performance could be assessed immediately by analyzing the patterns from these signals.

In this framework, the smart ball can be combined with any wearable device to provide a more complete measurement of ball-related skills. The locomotor performance and the cognitive function of each individual can be assessed by calculating the smart ball moving and rotating results and by monitoring the sensor patterns on the limb and the trunk. Moreover, consequential sports training can be adopted appropriately, according to individual performance.

This study aimed to examine object manipulation abilities, especially ball-related performance among adolescents with ID by using the smart ball and wearable sensors in four locomotor tests: two dribbling tests by foot (along a straight path and along a zigzag path with obstacles) and two ball-handling tests by hands (picking up a ball and throwing-and-catching).

The remainder of this paper is organized as follows. Section 2 shows the related works and the comparison with current systems in this research field. In Section 3, the methods of this study about the participants, materials, and parameters are described. In Section 4, the experimental results are reported. In Section 5, the analysis of the results and a discussion are provided. Lastly, the conclusion is presented in Section 6.

## 2. Related Works

Early studies used the optical motion capture system for motion detection [4,7,11,20,21,22,23]. This system can perform an accurate measurement and has been considered the gold standard for medical usage. However, the optical approach is very expensive and has time and space limitations in the test. Other previous studies have applied the wearable sensors based on microelectromechanical systems (MEMS) for motion detection [9,16,17,18,19]. They used the sensor signals to measure the temporal parameters, explored various abnormalities prior to formal assessment, or investigated physical activity levels in children and youth with ID [24]. This approach is very easy to use, but it is hard to measure the locomotor performance or cognitive function.

To date, there are very few studies about the smart ball/device designed for special needs population with alike features (recording, calculation, and analysis). The studies [25,26] adopted a smart ball (miCoach, Adidas, Herzogenaurach, Germany) with build-in motion sensors to measure the point of strike, speed, direction of spin, and flight path for training soccer kicks. These parameters were obtained by estimating the angle of kick and its initial velocity. However, people with ID cannot always perform a typical kick due to their poor motor performance. Therefore, this ball cannot perform a related analysis based on its designed algorithm. For use in people with ID, the parameters and the particular algorithm should be reconsidered based on their performance ability.

Table 1 summarizes the comparison of the optical motion capture system, wearable sensors, and the proposed smart ball. The smart ball approach can be more applicable to the measurement of locomotor performance and cognitive function with the advantages of low cost, low complexity, and ease of use.

## 3. Materials and Methods

### 3.1. Participants

The study was conducted in a special education school in Northern Taiwan. A total of 25 adolescents with ID aged 18.36 ± 2.46 years and 25 TD adolescents aged 18.36 ± 0.49 years participated in the study. Table 2 displays a summary of the age, gender, and sample size statistics of each group. Each adolescent with ID had one or more areas of significant impairment, such as orthopedic, autistic, or other neurodevelopmental disorders. The ability to perform activities of daily living (ADL) of each participant was screened by at least two physical therapists. ADL included transitioning from sitting to standing, transferring weight, obstacle crossing, selecting proper attire, dressing, and buttoning. A five-point score was used for each item with a score of 5 indicating independent functional performance. Participants with ID had a total score of 28.25 ± 1.83. A significant difference was identified between the two groups (ID and TD) in terms of ADL (*p* = 0.029). The adolescents with ID were able to follow simple commands and walk without support and had no serious heart or lung conditions. Each test was supervised by two physical therapists and an assistant to ensure safety. The participants and legal guardians signed informed consent forms. Chang Gung Memorial Hospital granted ethical approval for this study.

### 3.2. Measurement Devices

A nonwearable small ball and wearable motion trackers were used in the tests.

A. Nonwearable Smart Ball

**Figure 1:**
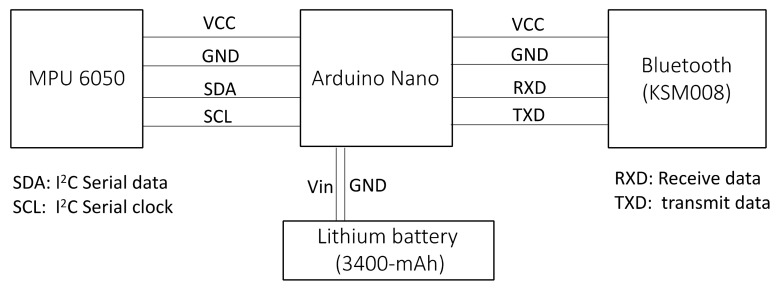
Illustration of the circuit design into the core of the ball.

The smart ball was fabricated with polyurethane (PU) foam material. An embedded circuit (Arduino Nano) with an integrated motion sensor (MPU-6050, InvenSense, CA, USA) and a Bluetooth adapter (KSM008, KAISE, Kaohsiung, Taiwan) was inserted into the core of the ball. The PU foam maintained the circuit tightly at the core of the ball to prevent the circuit from crashing from a collision. A 3400-mAh Lithium battery was attached to the circuit. The battery supported over 8 h of measurement on a full charge. The diameter of the ball was 20 cm and the total weight of the ball with the circuit and the battery was 510 g. The MPU-6050 is an integrated six-axis motion tracking device that combines a three-axis gyroscope, three-axis accelerometer, and a Digital Motion Processor (DMP) in a small package with a very low cost (about $10 USD). It also features three 16-bit analog-to-digital converters (ADCs) for digitizing the gyroscope outputs and three 16-bit ADCs for digitizing the accelerometer outputs [27]. The outputs from the core of the ball were wirelessly collected by a handheld computer with a 1 kHz sampling rate. A software application was designed to help the therapists collect and monitor the results in real-time. Figure 1 illustrates the circuit design of the core. Table 3 lists the major characteristics of the sensors in MPU-6050. The detailed specifications can be referenced in Reference [27].

B. Wearable: Xsens Tracker

Three Xsens MTw trackers (Xsens Technologies, Enschede, Netherlands) were secured to the outer sides of the arms, thighs, calves, and lower back for motion detection. Each Xsens tracker contained an integrated sensor (accelerometer, gyroscope, and magnetometer) that measured the rotation angles of the tracker in the sagittal, frontal, and transverse planes. Since the ball-related skill tests only involved forward and backward movements, only the angles on the sagittal plane were collected in this study. Each tracker was 47 × 30 × 13 mm^3^ (length × width × height) in size and weighed 16 g with a sampling rate of 1 kHz.

**Figure 2:**
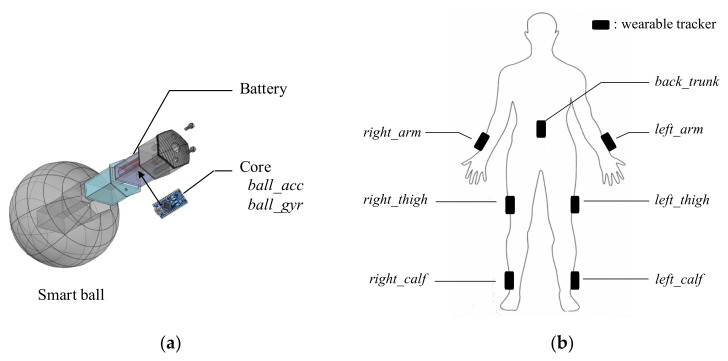
The location of the sensors: (**a**) the smart ball sensors, including an accelerometer (*ball_acc*) and a gyroscope (*ball_gyr*) at the core of the ball, (**b**) the wearable sensors, including six trackers at the right or left outer sides of the arms, thighs, calves, and one at the lower back.

Figure 2 illustrates the sensor positions, which were labeled *ball_acc*, *ball_gyr*, *left_arm*, *right_arm*, *left_thigh*, *right_thigh*, *left_calf*, *right_calf*, and *back_trunk* (“*acc*” represented the accelerometer, and “*gyr*” represented the gyroscope). All signals were collected by a handheld computer for analysis.

### 3.3. Testing Flow

Each participant completed two upper limb tests and two lower limb tests using the ball. In each test, the handheld computer played a prompt beep sound and the participant then started to perform the tests in the following order.

**Figure 3:**
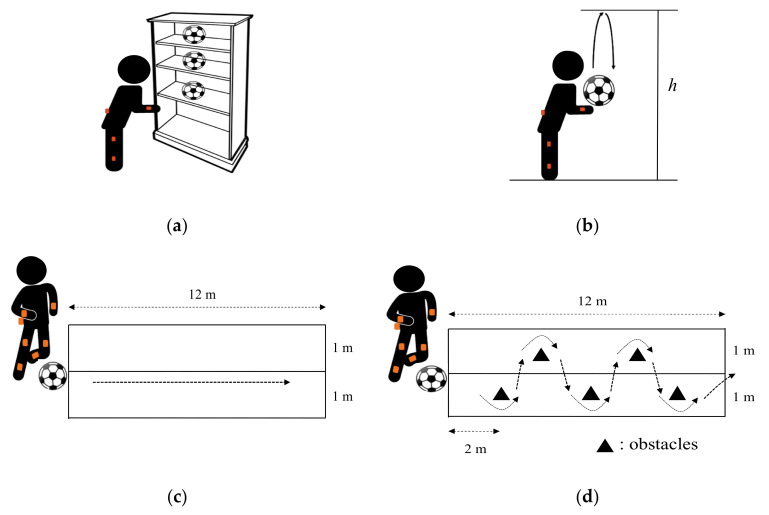
Illustrations of the tests: (**a**) picking up the ball at the spot it was placed, which was at three different heights, (**b**) throwing-and-catching the ball, where “*h*” denoted the height the ball should reach, (**c**) dribbling the ball along a straight line, where the dotted arrow showed the dribbling direction, and (**d**) dribbling the ball along a zigzag line with five obstacles staggered in a zigzag order.

(1) Picking up the ball: The ball was placed in front of the participant. After the beep sound, the participant was asked to pick up the ball as quickly as possible. The test repeated three times. The ball was placed at a different height each time (Figure 3a).
(2) Throwing and catching the ball: The participant first held the ball to the chest. After the beep sound, the participant was asked to throw up the ball vertically over a certain height (e.g., 2.5 m) and catch the ball stably with two hands (Figure 3b).
(3) Dribbling the ball along a straight line: The participant was asked to dribble the ball with the feet along a straight line in a lane that was 12 m in length and 2 m in width (1 m to each side of the straight line) without any obstacles (Figure 3c).
(4) Dribbling the ball with feet along a zigzag line: The participant was asked to dribble the ball with the feet along in the same lane (length 12 m, width 2 m) with five obstacles staggered in a zigzag order (Figure 3d).

Half of the students started from the upper limb tests (ball picking up, and throwing-and-catching), whereas the others started from the lower limb tests (dribbling along a straight line and a zigzag line). After completing these tests, the students switched. Three trials were performed for each test. The students rested for at least 1 min between tests.

The first two tests required participants to focus on the ball and use the arms to handle it without falling. Therefore, the abilities of rapid response and hand–eye coordination in an immediate event were measured by monitoring the parameters of each sensor in the tests. The final two tests relied more on the object-controlling ability. These tests required participants to maintain the trunk balance when moving the ball along a predefined path or around obstacles.

### 3.4. Parameters and Signal Processing

The following five parameters were obtained from the tests (Figure 3).

A. Average Reaction Time

($T_{reaction}$)

**Figure 4:**
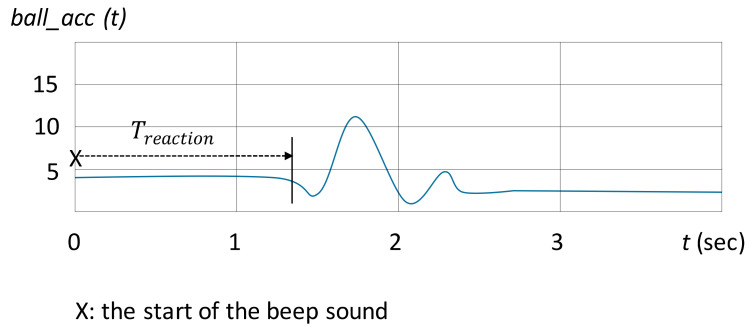
Illustration of the signal from *ball_acc*. The reaction time ($T_{reaction}$) was defined as the interval from the beep sound (denoteds as “x”) to the time at which the ball started moving.

In each test, the time interval from the beep sound to the time of which the ball started moving was defined as the reaction time. Figure 4 illustrates the signal captured from the sensor *ball_acc*. The starting time of the ball movement was detected by monitoring the rise or decline of the signal *ball_acc* from the initial state (Figure 4). The average reaction time was obtained by calculating the mean of the reaction times in all tests.

B. Throwing-and-Catching Rate

($R_{throwing-and-catching}$)

**Figure 5:**
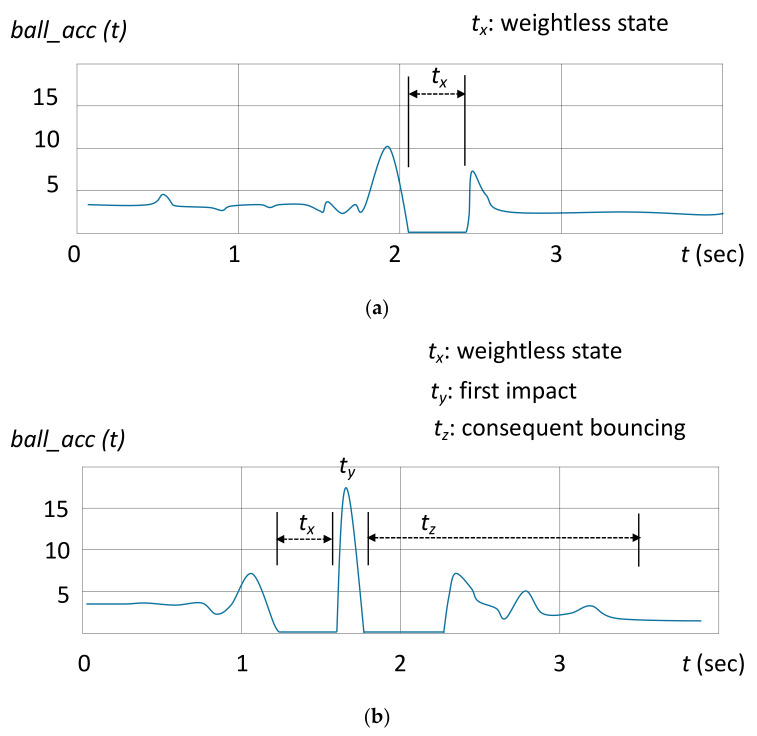
Illustrations of signals from *ball_acc*: (**a**) throw and catch successfully, where the signal was cut off (i.e., period “*t_x_*”) due to the weightless state as the sensor stays in the air, (**b**) throw without catching successfully, where “*t_y_*” was the first impact of the ball to the ground and was followed by another weightless period with a series of pulses due to the bounces of the ball (i.e., period “*t_z_*”).

This parameter was defined as the rate at which a participant threw the ball more than 2.5 m and caught it successfully among all trials. When the ball is thrown up, the sensor *ball_acc* has a short weightless period (*t_x_* in Figure 5a). In this period, the signal of *ball_acc* is cut-off. After the ball reaches the peak height at the center of this weightless period, it begins to fall. The peak height ($h\left(t\right)$) of a throw can be calculated as follows.

(1) $$h\left(t\right)=\frac{1}{2}g\;\cdot \;t^{2}$$

where t is the half time of the weightless period (i.e., *t_x_* in Figure 5a) and $g=9.8\;m/s^{2}$. In each test, a throw was considered successful when the value of $h\left(t\right)$ was higher than a certain distance (i.e., 2.5 m). After throwing, if the ball was caught without dropping to the ground, the catch was considered successful. The patterns of a successful catch can be distinguished by monitoring the signal *ball_acc.* In the case of a successful catch, a sharp pulse appears immediately after the weightless period, which is followed by a brief stable period (Figure 5a). In the case of an unsuccessful catch, the sharp pulse is followed by another weightless period and a series of pulses (i.e., *t_z_* in Figure 5b) due to the bounces of the ball.

C. Total dribbling time

($T_{dribbling\_straight},\;T_{dribbling\_zigzag}$)

**Figure 6:**
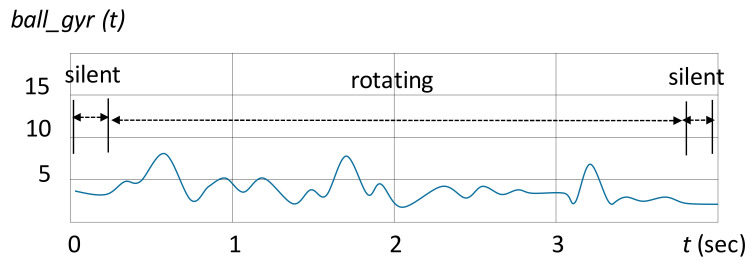
Illustration of the signal from *ball_gyr* during the straight-line dribbling. The total rotation angle of the ball can be obtained by accumulating the sensor signal during the rotating period.

This parameter measured the time from when a participant started to dribble to the time the ball reached the end and stopped rolling. In this interval, the starting time and the stopping time were detected separately by monitoring the signal from *ball_gyr* from the stable state to the rotating state and from the rotating state to the stable state, respectively. Figure 6 illustrates the signal of *ball_gyr* in the straight-line dribbling test. A baseline was established for detecting the signal as rising or falling.

D. Total Dribbling Distance

($D_{dribbling\_straight},\;D_{dribbling\_zigzag}$)

When the ball was dribbled on the ground, the internal sensor *ball_gyr* continued to rotate until stopping. Therefore, the dribbling distance of the ball was obtained by measuring the ball’s perimeter and counting the number of rotations (Equation (2)). The method used to derive the number of rotations was to accumulate the total rotation angle from the starting point to the stopping point and divide it by 360° $\left(2\pi \right)$. The rotation angle of the ball in a given time-period was obtained by calculating the integral of the rotation velocity (r/s) from the signal of sensor *ball_gyr.* Thus, the dribbling distance can be obtained as follows.

(2) $$D_{dribbling\_x}\;=\;\left(\frac{1}{2\pi }\int_{0}^{T_{dribbling\_x}}ball\_gyr\left(t\right)dt\right)*p_{ball},$$

where $x\in \left\{straight,\;zigzag\right\}$ and $p_{ball}$ is the perimeter of the ball.

E. Limb Swinging Angle

($A_{swing\_arm,}\;A_{swing\_thigh,}\;A_{swing\_calf,}$)

**Figure 7:**
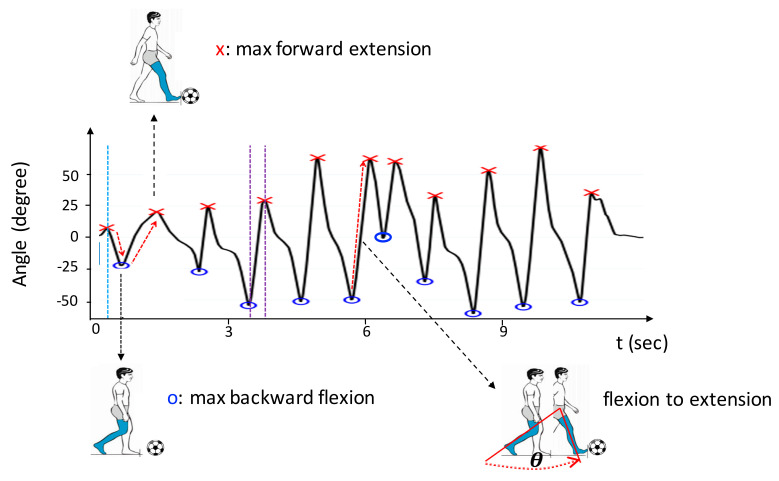
Illustration of the swinging angle measured from the *right_calf* on the sagittal plane. The angle from a valley point (blue circle) to a peak point (red cross) represented the degree through which the participant swung the calf once.

This parameter was defined as the angle through which a participant swung the arm, thigh, and calf to dribble the ball. Figure 7 displays a sample waveform of the rotation angle measured from the *right_calf* on the sagittal plane when a participant swung the right foot to dribble the ball. In this waveform, each peak point (marked with a red cross) represents the maximum extension degree of the calf moving forward, and each valley point (marked with a blue circle) represents the maximum flexion degree of the calf moving backward. Therefore, the angle from a valley point to a peak point represented the degree through which the participant swung the calf once (termed a swing) to move or dribble. The waveforms of the thighs and arms display similar patterns. Therefore, the average limb swinging angle of the arms ($A_{swing\_arm}$), thighs ($A_{swing\_thigh}$), and calves ($\;A_{swing\_calf}$) were obtained by measuring the average swinging angle of the left and right arms, the left and right thighs, and the left and right calves, respectively.

F. Limb Swinging Frequency

($F_{swing\_arm}$, $F_{swing\_thigh}$, $F_{swing\_calf}$)

In Figure 7, the period from a valley point to a peak point represented the time a participant swung the calf from flexion to extension. Therefore, the average limb swinging frequency of the arms ($F_{swing\_arm}$), thighs ($F_{swing\_thigh}$), and calves ($\;F_{swing\_calf}$) could be obtained by calculating the average swinging frequency of the left and right arms, left and right thighs, and left and right calves, respectively.

G. Trunk Tilt Angle

($A_{trunk}$)

**Figure 8:**
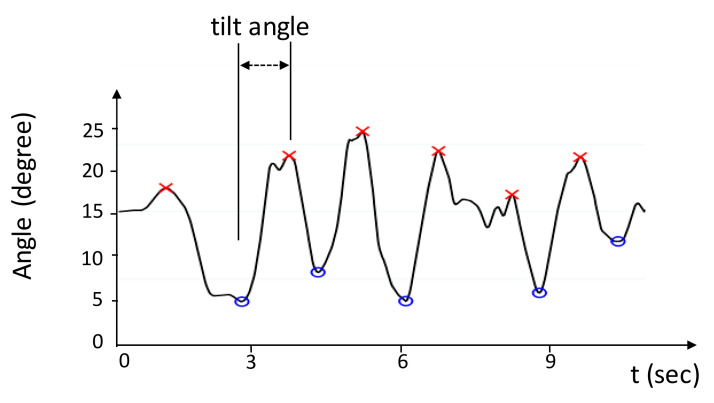
Illustration of the trunk tilt angle measured from *back_trunk* on the sagittal plane. The angle from a valley point (blue circle) to a peak point (red cross) represented the tilt angle through which the participant swung the trunk to maintain balance.

The trunk tilt angle was defined as the tilt angle of a participant’s trunk on the sagittal plane when dribbling the ball and moving. This parameter was obtained by monitoring the sensor *back_trunk* to test participants’ ability to maintain the trunk balance during the movement. Figure 8 displays a sample waveform of the trunk tilt angle on the sagittal plane.

### 3.5. Validation

Validation was performed by comparing the results of the wearable Xsens trackers and the smart ball with the Vicon system, which is a widely used optical motion capture device [28]. For each Xsens tracker, two rotation tests were applied including one from 0° to 90° and another from 0° to 180°. The bias of the tracker results compared with the Vicon output was used for calibration.

For the smart ball, six tests were applied: picking up the ball, throwing and catching the ball, and dribbling the ball over 5 m, 10 m, and 15 m. Four parameters were measured from the tests, including the reaction time for picking up a ball, throwing height, throwing-and-catching rate, and dribbling distance. The bias of the results after comparison were used for calibration.

**Figure 9:**
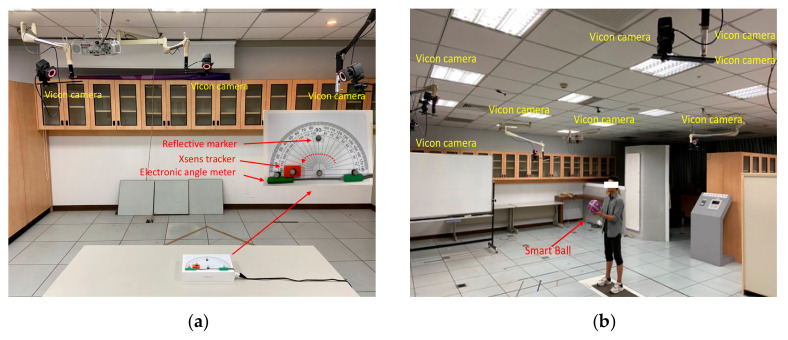
Illustrations of the validation tests: (**a**) for the Xsens tracker and (**b**) for the smart ball. All of the tests were captured under eight surrounded Vicon cameras.

Figure 9 shows how the validation tests were made. A platform was implemented with an electronic angle meter mounted on the top surface of it to confirm the testing angles (Figure 9a). Each Xsens tracker had one reflective marker pasted on it to synchronize the movement. The Xsens tracker rotated along with the angle meter and the reflective marker was detected by the Vicon cameras to report the track. For the smart ball, reflective tape was pasted on the surface of the ball to perform the ball playing tests (Figure 9b). All of the tests were captured under eight surrounded Vicon cameras.

## 4. Results

The experimental results comprised three parts: the validation results of the wearable trackers and the smart ball, the comparison of the ball-related skill performance between adolescents with ID and TD adolescents, and the comparison of the limb and trunk parameters between the two groups during tests.

### 4.1. Validation Results

A. Xsens Tracker Validation

Table 4 displays the validation results of the trackers placed on the *arm*, *thigh*, *calf*, and *back_trunk* in the 90° to 180° rotation test. The results revealed that the reported degrees of all trackers were higher than the Vicon results. In the 90° test, the bias ranged from 3.0% to 4.1%, and, in the 180° test, it ranged from 2.9% to 3.4%. This finding revealed a static error in the rotation measurement for each Xsens tracker used in this study.

B. Smart Ball Validation

Table 5 displays the validation results of the small ball. The first three parameters (reaction time, throwing height, and throwing-and-catching rate) were obtained from picking up the ball test and the throwing and catching test. The results revealed that the smart ball had 3.1% and 2.9% error rates in measuring the time and height. In the ball throwing-and-catching test, the smart ball displayed only one error out of 60 trials. This finding indicated that the smart ball identified a successful throw and catch with a very low error rate (1.6%).

The final parameter (dribbling distance) was the distance over three distances (5, 10, and 15 m). The error rates of the smart ball for these distances were 2.6%, 3.7%, and 1.12%, respectively. These errors were principally caused by the drift effects of the sensors [29,30]. Therefore, the sensor results were calibrated using these errors in the following tests.

### 4.2. Ball-Related Skill Performance

A. Average Reaction Time

**Figure 10:**
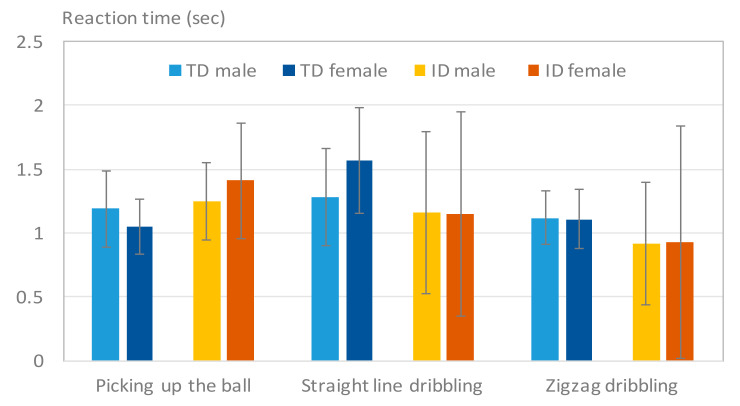
Results of $T_{reaction}$. The participants with an intellectual disability (ID) had a slower reaction time only in the picking-up-the-ball test.

Figure 10 illustrates the reaction time of the participants with ID and TD participants in three different tests. The reaction times of the ID participants were not always slower than the TD participants in each test. Take Figure 10 for example. The ID participants had, on average, a longer reaction time in the picking-up-the-ball test, but a shorter reaction time in the dribbling test when compared with the TD participants. One possible reason for this is that the motion of dribbling the ball is more straightforward, and easier to follow the order than the motion of picking up the ball for the ID participants. Thus, when the ball was placed on the ground, most of them can start to dribble the ball quickly. However, when the ball was placed on different heights, they had to think about the height and tried to use hands to pick up the ball. It would significantly increase their reaction time. Table 6 lists the analysis of variance (ANOVA) of the TD adolescents and the adolescents with ID in these three tests. The results indicated that the difference between the two groups in the reaction time for the picking-up-the-ball test was statistically significant, but not for the two dribbling tests. The *p*-values in Table 6 shows that this difference was not always statistically significant because some of the ID participants still displayed very poor motor performance.

TD girls and boys exhibited significantly different results in picking up the ball and straight-line dribbling tests, whereas girls and boys with ID only differed in the picking-up-the-ball test. No basic difference was observed between the reaction times of boys and girls in ball-related skills. However, girls with ID exhibited greater variations than boys with ID in the tests.

B. Successful Throwing-and-Catching Rate

**Figure 11:**
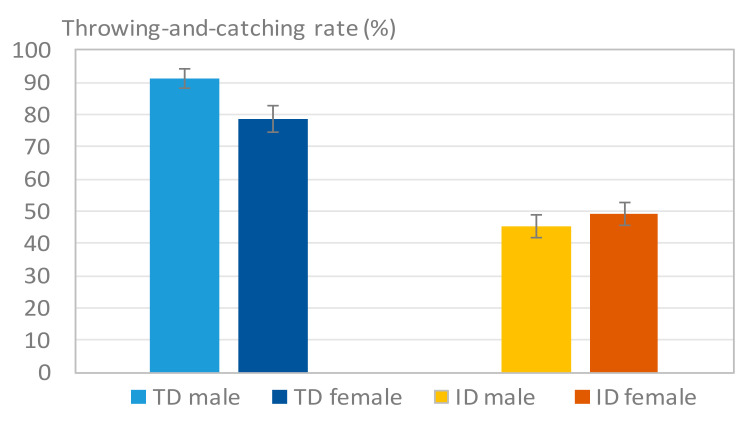
Results of $R_{throwing-and-catching}$. The paticipants with ID exhibited clearly lower throwing-and-catching rates than the typically developing (TD) participants.

Figure 11 displays the throwing-and-catching rates for the TD participants and the participants with ID. The results indicated a significant difference between the two groups. The average throwing-and-catching rate was >75% in the TD group, whereas the highest rate did not exceed 55% in the ID group. This finding indicates that this test presents a challenge for participants with ID. Only approximately half of the participants with ID could perform this task. Table 7 lists the mean values of the TD and ID groups in this test. The ANOVA results revealed that the difference between the two groups (*p* = 0.001) was highly significant.

C. Total Dribbling Distance

**Figure 12:**
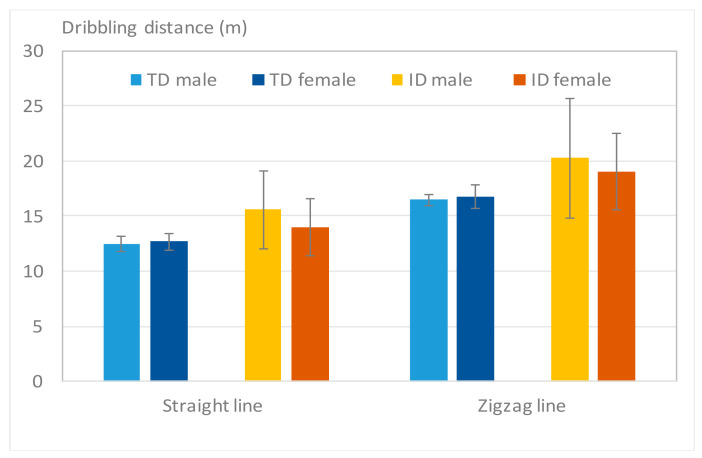
Results of $D_{dribbling\_straight}$ and $D_{dribbling\_zigzag}$. The participants with ID exhibited longer dribbling distances than the TD participants in both tests.

Figure 12 displays the average dribbling distance performed by adolescents with TD and ID in the straight-line and zigzag-line tests. Shorter dribbling distances were considered to enable gesture adjustment and a more accurate finish of the dribbling. Notably, participants with ID exhibited longer dribbling distances than the TD participants in both of the tests. In the straight-line test, TD participants required 12.59 m to finish the test, whereas participants with ID required 14.70 m (Table 8). Some boys with ID exceeded 15 m. In the zigzag test, the difference in the average dribbling distance between two groups increased (i.e., 16.62 m versus 19.76 m). The ANOVA results revealed that the differences between the TD and ID groups were highly significant in both the straight-line and zigzag-line tests (*p* < 0.01, Table 8). This finding indicated that the participants with ID generally dribbled the ball with more bias away from the preset path with a higher chance of being out-of-bounds. Consequently, individual dribbling performances among the participants with ID displayed a wider variety when compared with the TD participants (Figure 12).

D. Total Dribbling Time

**Figure 13:**
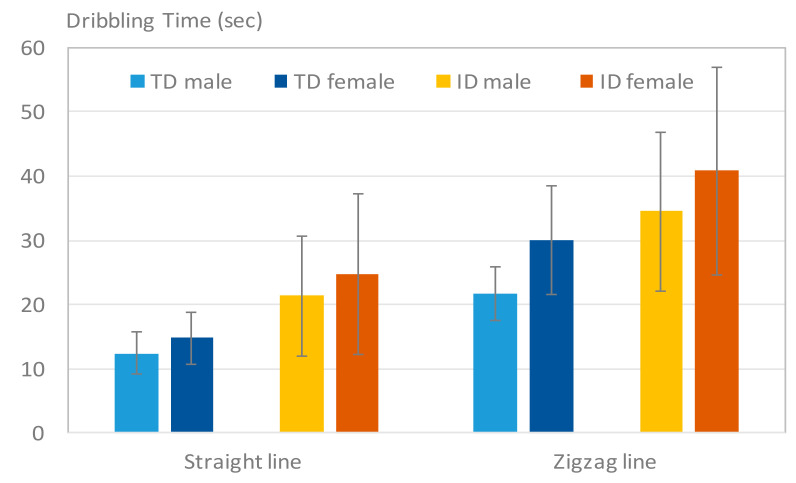
Results of $T_{dribbling\_straight}$ and $T_{dribbling\_zigzag}$. The participants with ID displayed longer dribbling times than the TD participants in both tests.

Figure 13 illustrates the average dribbling time of the TD participants and participants with ID in the straight-line and zigzag-line tests. Shorter dribbling times suggested that the subjects controlled the ball more adaptably. Similar to the dribbling distance, participants with ID required more time on average to dribble the ball along the paths. However, participants with ID required almost double the time when compared with the TD participants. The individual variation was also larger in both the straight-line and zigzag-line tests. Moreover, girls with ID exhibited the longest dribbling times in all tests.

Table 9 displays the ANOVA results of the dribbling time. The difference between the two groups of participants was statistically significant in both paths.

### 4.3. Limbs and Trunk Performance

A. Limb Swinging Frequency

**Figure 14:**
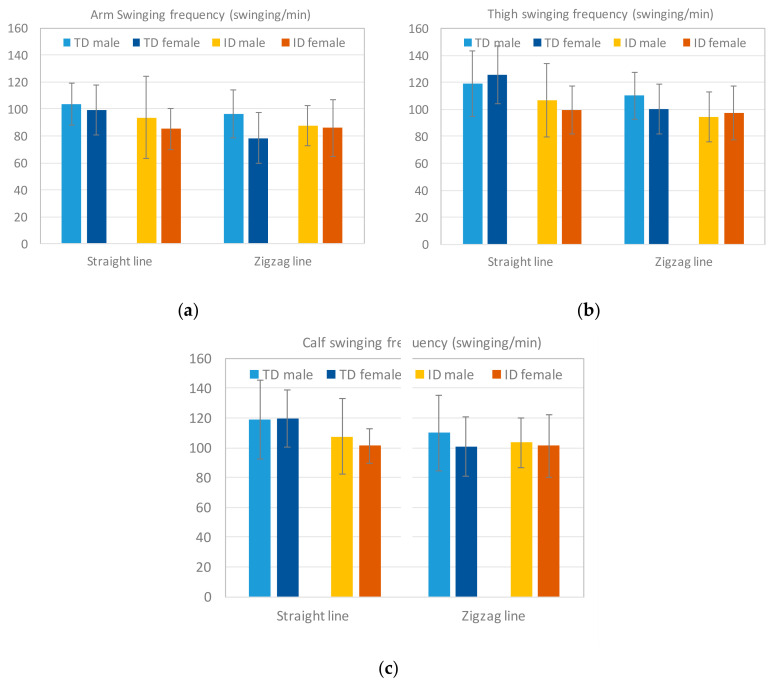
Results of the limb swinging frequency: $\left(a\right)\;F_{swing\_arm},\left(b\right)\;F_{swing\_thigh},\;\mathrm{and}\;\left(c\right)\;F_{swing\_calf}.$ The participants with ID displayed overall lower limb swing frequencies in both tests.

The limb motions of the arms, thighs, and calves were observed in the dribbling tests. Figure 14 displays the average swinging frequency of each pair of limbs in TD participants and participants with ID. A clear difference was observed between groups for each pair of limbs in the straight-line tests with the ID group displaying lower swinging frequencies. In the zigzag-line tests, although the gap of the swinging frequency of each pair of limbs was not as large as the difference in the straight-line tests, the TD participants displayed faster limb swinging on average. This result can explain why overall longer dribbling times were observed among participants with ID (Figure 13) in both the straight-line and zigzag-line tests. The ANOVA results revealed significant differences between the two groups in terms of the swinging frequencies of the thighs and calves in the straight-line tests (Table 10).

B. Limb Swinging Angle

**Figure 15:**
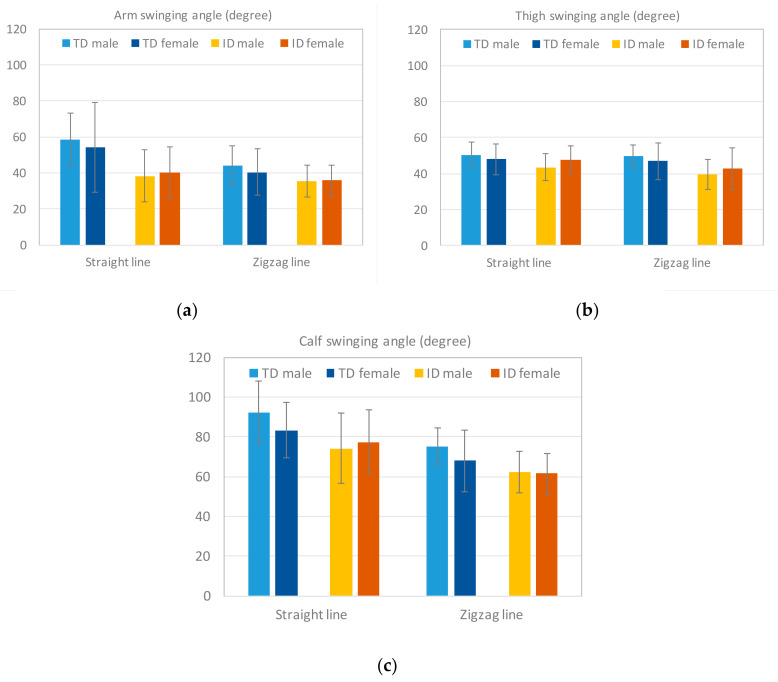
Results of the limb swinging angle: $\left(a\right)\;A_{swing\_arm},\left(b\right)\;A_{swing\_thigh},\mathrm{and}\;\left(c\right)\;A_{swing\_calf}$. The participants with ID showed overall smaller limb swing angles in both tests, where the difference of the calf angles reached more than 10°.

The limb swinging angle is another crucial indicator of limb coordination in the dribbling motion. Figure 15 displays the average swinging angle of the arms, thighs, and calves of participants, measured in the straight-line and zigzag-line tests. The results reveal that TD participants swung the limbs with a larger angle than participants with ID when dribbling the ball. The difference between the two groups reached more than 10° (Table 11). This finding illustrates that the participants with ID adjusted the limbs by using slower and less smooth postures to maintain balance for the same motion. Table 11 also reveals significant differences in the calf and the arm, which are the two body parts that control the ball along the paths.

C. Trunk Tilt Angle

**Figure 16:**
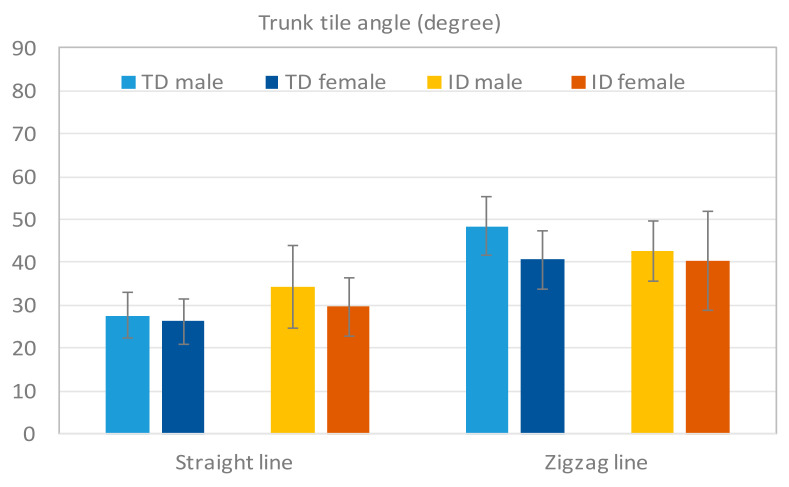
Results of the trunk tilt angle $A_{trunk}$. The participants with ID displayed larger tilt angles in the straight-line test but lower angles in the zigzag line test.

The trunk tilt angle can be used to observe how an individual bent the trunk to maintain the balance during movement. Figure 16 displays the trunk tilt angles of the TD participants and participants with ID during the dribbling tests. In the straight-line test, both the boys and girls with ID exhibited higher trunk tilt angles than did the TD boys and girls, whereas only the TD boys presented a larger tilt angle than the boys with ID in the zigzag-line test. The girls with ID and TD girls did not exhibit significant differences in this test. The ANOVA results revealed significant differences in the straight-line test (*p* < 0.05, Table 12).

## 5. Discussion

Several aspects of motor performance differed significantly between TD participants and participants with ID. First, participants with ID displayed worse upper limb performance when compared with the TD participants. In the picking-up-the-ball tests, the participants with ID had an average reaction time of 1.3 s, whereas the reaction time of TD participants was only 1.1 s. Moreover, participants with ID were successful in approximately half of the catches (46.8%) in the throwing-and-catching test, which also involved upper limb function, whereas TD participants achieved a success rate of more than 80.0%. Throwing and catching a ball requires integrated motor function, such as flexible joint motion, sufficient arm force, good hand–eye coordination, and balance of the trunk. This result illustrates that participants with ID generally demonstrated poor integration of motor function when compared with TD participants.

Second, the participants with ID also exhibited less favorable locomotor performance of the lower limbs than TD participants. In both the straight-line test and the zigzag-line test, the participants with ID required a longer time to dribble and dribbled in a less efficient path than TD participants, which resulted in a longer distance before completion. This result indicated that ball dribbling is a challenging task for participants with ID, which may be because it requires integrated lower extremity strength, cardiopulmonary endurance, vision, balance, and object control. Other performance features were observed in the participants with ID. First, the dribbling speed was slower than TD participants. Second, the lower extremity control was insufficient to roll the ball precisely along the requested path. Third, the dribbling performance was relatively unstable, and they sometimes used the hands to stop the ball from rolling out of the path.

Participants with ID also displayed significant differences in terms of limb movement patterns during the dribbling test when compared with TD participants. The results revealed that participants with ID had lower swing frequencies and smaller swing angles for both upper and lower limbs. This finding indicated that participants with ID used small and slow steps to move. Moreover, participants with ID had a higher trunk tilt angle than TD participants, which indicated that they used a forward posture during dribbling to prevent falls. These patterns are difficult to observe in normal walking tests but were demonstrated when using our design.

The results in this study also revealed that the measurements from the wearable sensors and the smart ball were highly correlated. The smart ball was proposed to measure object controlling performance. Compared with the wearable sensors, which mainly measure the motions of the extremity limbs and trunk, the smart ball can be used to evaluate performance outcomes without the limitations associated with wearing sensors. Therapists and teachers could evaluate an individual’s motor performance from different perspectives by combining wearable sensors with the smart ball. For instance, the participants with ID used a slow and short gait to move the ball during dribbling, which could have caused poor dribbling performance. This finding can be applied to facilitate differential diagnosis and to monitor training progress in adolescents with ID.

The smart ball was developed by embedding the inertial sensors into the core. The embedded algorithm was specifically designed for the individuals with ID based on their motor characteristics and level of understanding. The designed software was able to collect and monitor the moving and rolling data automatically and continuously by using wireless communication. Most of all, the ball-related tests were attractive to participants with ID, which would increase their willingness to perform the requested evaluation or training tasks.

To date, very few current existing devices can perform the desired function. The use of this ball for motor performance evaluation in individuals with ID is a breakthrough in measurement technology. Compared with the traditional expert scoring tools, instead of an overall rough description of ball skill performance, it can provide detailed performance parameters, such as reaction time, dribbling time, dribbling distance, and a successful rate objectively and efficiently. It is also suitable for mass screening in schools and institutions where the population need to be evaluated in a short period of time such as admission assessment. Through this smart ball, plenty of manpower can be saved, which makes the whole process more time-saving and effective. This smart technology can be applied to other evaluation tools and devices in the future.

## 6. Conclusions

In this study, a smart ball was developed to evaluate the locomotor performance of adolescents with ID. The results revealed that this approach discriminated between TD participants and participants with ID in terms of reaction time, throwing-and-catching rate, and dribbling performance along different paths. Performance evaluations could be extended to numerous aspects of motor control by designing different ball-related tasks. This study also demonstrated that the combination of the wearable sensors and the nonwearable sensors (i.e., the smart ball) can increase the objectivity, comprehension, and convenience of locomotor evaluation for large groups of participants.

## Figures and Tables

**Table 1:** Comparison of the proposed smart ball with the optical motion tracking system and the wearable trackers.

Requirement	Optical Motion Capture System	Wearable Sensors	Proposed Smart Ball
Motion tracking	Applicable	Applicable	Partially applicable
Locomotor performance	Partially applicable	Partially applicable	Applicable
Cognitive ability	Inapplicable	Inapplicable	Applicable
Complexity	High	Medium to low	Low
Cost	$10,000–$20,000	$1000–$10,000	$1000–$2000

**Table 2:** Summary of the age, gender, and sample size for the ID and TD groups. Both groups had the same number of participants and an equal range of the age.

Group	Total	Age (Mean ± “SD”)	Gender	Number	Age (Mean ± “SD”)
ID	25	18.36 ± 2.46	Male	15	18.00 ± 2.56
			Female	10	18.90 ± 2.33
TD	25	18.36 ± 0.49	Male	11	18.55 ± 0.52
			Female	14	18.21 ± 0.43

**Table 3:** Characteristics of the sensors in MPU-6050.

Sensors	Parameter	Type
gyroscope	Voltage (VDD)	1.8 V, 5 V
	Full-Scale Range	±250 °/s, ±500 °/s, ±1000 °/s, ±2000 °/s
	ADC Word Length	16 bits
	Nonlinearity	Best fit straight line, 25 °C
	Frequencies (x, y, z)	33 KHz, 30 KHz, 27 KHz
	Start-up time	30 ms
accelerometer	Voltage (VDD)	1.8 V, 5 V
	Full-Scale Range	±2 g, ±4 g, ±8 g, ±16 g
	ADC Word Length	16 bits
	Nonlinearity	Best fit straight line; 25 °C
	Initial Calibration Tolerance (x, y, z)	±50 mg, ±80 mg, ±35 mg
	Start-up time	30 ms

**Table 4:** Validation results of the trackers in the 90° and 180° rotation tests. The results showed that each tracker has a static error rate <4%.

	Rotation of 90°			Rotation of 180°		
Sensor	Tracker (°)	Vicon (°)	Error (%)	Tracker (°)	Vicon (°)	Error (%)
*arm*	93.41	90	3.8	185.65	180	3.1
*thigh*	92.98	90	3.3	185.34	180	2.9
*calf*	92.72	90	3.0	185.46	180	3.0
*back_trunk*	93.71	90	4.1	186.12	180	3.4
Mean	93.21	90	3.6	185.64	180	3.1

**Table 5:** Validation of the smart ball in the three tests. The results showed that the ball had stable error rates in measuring all parameters.

Parameter	Smart Ball	Vicon	Error Rate
Reaction time	3.57 (s)	3.45 (s)	3.1 (%)
Throwing height	1.16 (m)	1.14 (m)	2.9 (%)
Throwing-and-catching rate (success/total)	29/60	30/60	1.6 (%)
Dribbling distance (5 m)	5.13 (m)	–	2.60 (%)
Dribbling distance (10 m)	10.37 (m)	–	3.70 (%)
Dribbling distance (15 m)	15.17 (m)	–	1.12 (%)

**Table 6:** ANOVA results of the reaction time for the two groups in three tests.

Test	TD Mean ± SD (s)	ID Mean ± SD (s)	*p*-Value
Picking-up-the-ball	1.11 ± 0.26	1.31 ± 0.36	0.032 *
Dribbling (Straight-line)	1.44 ± 0.42	1.16 ± 0.69	0.079
Dribbling (Zigzag)	1.11 ± 0.22	0.92 ± 0.66	0.185

* Mean difference is significant at *p* < 0.05.

**Table 7:** ANOVA results of the throwing-and-catching rate for the two groups.

Test	TD Mean ± SD (s)	ID Mean ± SD (s)	*p*-Value
Throwing-and-catching	84.0 ± 3.74	46.8 ± 3.59	0.001 *

* Mean difference is significant at *p* < 0.05.

**Table 8:** ANOVA results of the dribbling distance for the two groups.

	TD Mean ± SD (s)	ID Mean ± SD (s)	*p*-Value
Dribbling (Straight-line)	12.59 ± 0.73	14.70 ± 3.24	0.001 *
Dribbling (Zigzag-line)	16.62 ± 0.85	19.76 ± 4.71	0.002 *

* Mean difference is significant at *p* < 0.05.

**Table 9:** ANOVA results of the dribbling time for the two groups.

Test	TD Mean ± SD (s)	ID (sec) Mean ± SD (s)	*p*-Value
Dribbling (Straight-line)	13.74 ± 3.88	22.72 ± 10.60	0.000 *
Dribbling (Zigzag-line)	26.35 ± 7.96	37.01 ± 14.05	0.002 *

* Mean difference is significant at *p* < 0.05.

**Table 10:** ANOVA results of the limb swinging frequency (swings/minute) in the two groups.

	Arm			Thigh			Calf		
Test	TD M ± SD	ID M ± SD	*p*	TD M ± SD	ID M ± SD	*p*	TD M ± SD	ID M ± SD	*p*
Straight Dribbling	101.25 ± 16.95	90.20 ± 25.40	0.077	122.76 ± 22.49	103.94 ± 23.69	0.006 *	119.37 ± 22.19	105.10 ± 21.14	0.024 *
Zigzag Dribbling	86.23 ± 20.14	86.26 ± 17.16	0.898	104.56 ± 18.28	95.63 ± 18.68	0.094	104.90 ± 22.46	99.36 ± 16.63	0.229

* The mean difference is significant at *p* < 0.05.

**Table 11:** ANOVA results of limb (arm, thigh, and calf) swinging angle in the two groups.

	Arm Swinging Angle			Thigh Swinging Angle			Calf Swinging Angle		
Test	TD M ± SD	ID M ± SD	*p*	TD M ± SD	ID M ± SD	*p*	TD M ± SD	ID M ± SD	*p*
Straight Dribbling	56.02 (±20.74)	39.05 (±14.13)	0.001 *	49.03 (±7.82)	45.27 (±7.73)	0.094	87.14 (±15.06)	75.35 (±16.87)	0.012 *
Zigzag Dribbling	41.97 (±11.87)	35.68 (±8.55)	0.037 *	47.39 (±8.73)	40.89 (±9.47)	0.009 *	71.07 (±13.38)	61.88 (±10.12)	0.009 *

* The mean difference is significant at *p* < 0.05.

**Table 12:** ANOVA results of the trunk tilt angle in the two groups.

Test	TD (sec) (Mean ± SD)	ID (sec) (Mean ± SD)	*p*-Value
Dribbling (Straight-line)	26.77 ± 5.27	32.38 ± 8.81	0.009 *
Dribbling (Zigzag-line)	44.05 ± 7.66	41.69 ± 8.95	0.322

* The mean difference is significant at *p* < 0.05.

## Abbreviations

AAIDD	American Association of Intellectual and Developmental Disabilities
ID	Intellectual disability
TD	Typical developing
PDMS-2	Peabody Developmental Motor Scales, Second Edition
PGMQS	Preschooler Gross Motor Quality Scale
PU	Polyurethane
